# Canadian Expert Recommendations on Safety Overview and Toxicity Management Strategies for Sacituzumab Govitecan Based on Use in Metastatic Triple-Negative Breast Cancer

**DOI:** 10.3390/curroncol31090422

**Published:** 2024-09-21

**Authors:** Mita Manna, Michelle Brabant, Rowen Greene, Michael Dean Chamberlain, Aalok Kumar, Nimira Alimohamed, Christine Brezden-Masley

**Affiliations:** 1 Department of Medicine, University of Saskatchewan, Saskatoon, SK S7N 5A2, Canada; 2 Department of Oncology, College of Medicine, University of Saskatchewan, Saskatoon, SK S7N 5E5, Canada; 3 Saskatoon Cancer Centre, Saskatchewan Cancer Agency, Saskatoon, SK S7N 4H4, Canada; 4 Department of Biochemistry, Microbiology, and Immunology, College of Medicine, University of Saskatchewan, Saskatoon, SK S7N 5E5, Canada; 5 BC Cancer Surrey, University of British Columbia, Surrey, BC V3V 1Z2, Canada; 6 Department of Medicine, University of Calgary, Calgary, AB T2N 4N2, Canada; 7 Department of Oncology, University of Toronto, Toronto, ON M5S 1A1, Canada

**Keywords:** sacituzumab govitecan, antibody-drug conjugate, triple-negative breast cancer, adverse event management

## Abstract

Sacituzumab Govitecan (SG) is an antibody-drug conjugate (ADC) comprised of an anti-Trop-2 IgG1 molecule conjugated to SN-38, the active metabolite of irinotecan, via a pH-sensitive hydrolysable linker. As a result of recent Canadian funding for SG in advanced hormone receptor (HR)-positive breast cancer and triple-negative breast cancer (TNBC), experience with using SG and managing adverse events (AEs) has grown. This review presents a summary of evidence and adverse event recommendations derived from Canadian experience, with SG use in metastatic TNBC for extrapolation and guidance in all indicated settings. SG is dosed at 10 mg/kg on day 1 and day 8 of a 21-day cycle. Compared to treatment of physicians’ choice (TPC) the phase III ASCENT and TROPiCS-02 studies demonstrated favorable survival data in unresectable locally advanced or metastatic TNBC and HR-positive HER2 negative metastatic breast cancer, respectively. The most common AEs were neutropenia, diarrhea, nausea, fatigue, alopecia, and anemia. This review outlines AE management recommendations for SG based on clinical trial protocols and Canadian guidelines, incorporating treatment delay, dose reductions, and the use of prophylactic and supportive medications.

## 1. Introduction

Triple-negative breast cancer (TNBC) is a biologically aggressive form of breast cancer defined by the absence (0 or ≤1% immunohistochemistry (IHC) expression) of the hormone receptors (HR) estrogen (ER) and progesterone (PR), as well as a lack of amplification of the human epidermal growth factor receptor 2 (HER2), accounting for up to 20% of breast cancers [1].

Metastatic TNBC (mTNBC) is a heterogeneous disease with few treatment options and poor outcomes, mainly due to a lack of targetable receptors [2,3,4,5]. Until recently, chemotherapy remained the standard of care treatment option for patients with TNBC, despite being associated with higher and early risk of recurrence, short survival, low response rates (RR), and significant toxicity [6,7,8].

The combination of limited treatment options with aggressive tumor biology and treatment-related toxicities highlights the need to identify novel systemic treatment options to improve outcomes for patients with mTNBC. Antibody-drug conjugates (ADCs) are drugs that consist of monoclonal antibodies chemically linked to highly potent cytotoxic drugs. ADCs first bind to a specific target protein on the tumor cell via its monoclonal antibody [9]. The ADC is then internalized, which enables the cleavage of the linker, thereby releasing the cytotoxic drug into the cell [9]. Unlike most standard chemotherapies, the ability to selectively target malignant cells has made ADCs a popular approach to cancer therapeutics [9]. To date, three ADCs have been approved by Health Canada for the treatment of breast cancer, two of which are anti-HER2 ADCs. The first, trastuzumab emtansine (T-DM1), is approved for treating early and metastatic HER2-positive breast cancer [10,11]. The second, trastuzumab deruxtecan (T-DXd), was first approved to treat heavily pretreated HER2-positive mBC [12], and later for the treatment of pretreated HER2-low advanced breast cancer [13]. HER2-low disease was defined as a score of 1+ in IHC analysis or an IHC score of 2+ and negative results in in situ hybridization (ISH) [14,15].

The third ADC that is approved in Canada for mBC is sacituzumab govitecan (SG), which targets trophoblast cell surface antigen-2 (Trop-2) through a humanized monoclonal anti-Trop-2 IgG1 (clone hRS7 IgGlk) and releases SN-38 as a cytotoxic payload [16,17]. SN-38 is the active metabolite of irinotecan and acts as a topoisomerase 1 (TOPO1) inhibitor. In SG, SN-38 is conjugated at a high drug-to-antibody ratio (DAR) of 7.6:1 via a pH-sensitive proprietary hydrolysable linker CL2A [18,19,20]. The high DAR of SG permits the release of a high SN-38 concentration without negatively affecting binding or pharmacokinetics [16,18]. The hydrolysable linker is designed to liberate cytotoxic SN-38 in the acidic tumor microenvironment without prerequisite internalization and subsequent enzymatic cleavage [21], enabling “bystander effect” tumor cell killing [22], as free SN-38 is membrane-permeable [20]. Please see Supplementary Materials for Pharmacology Overview of SG, which includes references [23,24,25,26,27].

Health Canada granted a Notice of Compliance (NOC) to SG in September 2021 for treating adult patients with unresectable locally advanced or mTNBC who had received two or more prior therapies [17]. On 19 July 2023, this indication was expanded to include adult patients with unresectable locally advanced or metastatic HR-positive, HER2-negative or low breast cancer who have received endocrine-based therapy and at least two additional systemic therapies in the metastatic setting [17].

SG is the first and only approved ADC for both mTNBC and metastatic HR-positive, HER2-negative/low breast cancer and the first approved ADC to target Trop-2 [16]. Trop-2 is a transmembrane glycoprotein known as a tumor-associated calcium signal transducer [28]. Its overexpression is associated with poor prognosis and increased tumor growth in various solid tumors, including breast, colorectal, cervical, esophagus, gastric, lung, pancreatic, and prostate cancers [16,29,30]. In TNBC, Trop-2 is highly expressed in 96% of patients, making it an ideal therapeutic target [16]. Given the high expression of Trop-2 in breast cancers and other epithelial cancers, and the data showing responses regardless of Trop-2 expression, pre-therapy biomarker assessment for SG treatment is not considered beneficial [31].

Although SG was approved in Canada for patients with mTNBC in 2021, it was not publicly reimbursed until 2023, leading to limited clinical experience with its use and associated toxicity management. To maximize SG treatment’s efficacy and decrease the incidence of significant AEs, proper evaluation and care are essential. This review aims to present professional insights from medical oncologists across Canada on managing AEs with SG, based on experience in the mTNBC setting. With SG’s recent approval in 2024 for HR-positive, HER2-negative/low metastatic breast cancer (though not yet funded), these clinical experiences may soon be applicable to this expanding indication. In this review, an evaluation was conducted of key clinical trials, including IMMU-132-01, ASCENT, and TROPiCS-02 [23,31,32]. Section 2 provides a comprehensive overview of the results from each these studies. In Section 3 and Section 4, clinical guidelines and reports from the pharmaceutical company are utilized to outline recommendations by Canadian experts for managing side effects associated with the use of SG.

## 2. Summary of SG Clinical Evidence

SG was first analyzed in the phase I/II IMMU-132-01, a single-arm multicohort study evaluating the safety and tolerability of SG as a single agent in previously treated metastatic epithelial cancers. The TNBC cohort included 108 patients who had received a median of three previous therapies. These patients received SG at a dose of 10 mg/kg, which resulted in an overall response rate (ORR) of 33.3%, a median PFS of 5.5 months, a median OS of 13 months, and a good therapeutic index. Given the ORR and therapeutic index, a dose of 10 mg/kg was chosen as the preferred dose for future development [23].

The results from IMMU-132-01 led to the phase III ASCENT study, which was a global, open-label randomized study to evaluate the efficacy, safety, tolerability, and pharmacokinetics (PK) of SG versus treatment of physician’s choice (TPC; eribulin, capecitabine, vinorelbine or gemcitabine) in patients with unresectable locally advanced or mTNBC, refractory or relapsed after two prior standard-of-care chemotherapy regimens [20]. A hazard ratio (HR) for PFS of 0.41 (N= 468; 95% CI 0.32–0.52; *p* < 0.001) was observed. The median PFS was 5.6 months for SG versus 1.7 months for TPC. For the secondary endpoint OS, a HR of 0.48 (N = 468; 95% CI 0.38–0.59; *p* < 0.001) was observed. The median OS was 12.1 months versus 6.7 months, in patients treated with SG and TPC, respectively. A post hoc sub-analysis of SG in the intention to treat (ITT) population of mTNBC showed therapeutic benefit, whether it was HER2-negative defined by IHC 0 or HER2-low [23,26].

TROPiCS-02 was another phase III trial evaluating the use of SG versus chemotherapy in pretreated, endocrine-resistant HR-positive, HER2-negative mBC [32]. SG therapy resulted in an ORR of 21% vs. 14% (OR 1.63, 95% CI, 1.03–2.56, *p* = 0.035), a median PFS of 5.5 months (95% CI, 4.2–7.0) vs. 4.0 months (95% CI, 3.1–4.4), a median OS of 14.4 months vs. 11.2 months (HR 0.70, 95% CI 0.65–0.96, *p* = 0.020), and a CBR of 34% vs. 22% when compared to the standard TPC chemotherapy [31,33]. A comparison of the efficacy data from these studies is shown in Table 1.

## 3. Safety Overview

The prevalence of AEs resulting from SG treatment reported here is derived from patient data from the ASCENT (*n*= 258) and TROPiCS-02 randomized controlled trials (*n* = 268), in which patients received at least one dose of SG as monotherapy [26]. Although no deaths within the SG arm of the ASCENT study were deemed to be treatment-related, severe AEs that required preliminary treatment discontinuation occurred in 5% of patients in both the TPC and SG groups [20]. The most common AEs leading to the discontinuation of SG were pneumonia (1%) and fatigue (1%) [20]. The most common AEs of any grade experienced during SG treatment in ASCENT and TROPiCS-02 (N = 526) included neutropenia (66.7%), diarrhea (58.0%), nausea (56.1%), fatigue (41.1%), alopecia (46.0%), anemia (34.2%), and vomiting (24.0%) [20,31].

The safety population within the ASCENT trial specifically included 482 patients who received at least one treatment dose (258 in the SG arm and 224 in the TPC arm). Table 2 displays the prevalent treatment-related adverse events (TRAE) of all levels and those specifically of grade ≥3, including neutropenia, diarrhea, and nausea. Dose reductions were required in 22% of patients within the SG arm and in 26% of patients receiving TPC [25]. However, the SG arm was more prone to severe AEs that required a dose interruption in 61% of patients, compared to 33% in the TPC arm [25].

The dose of SG should be modified in accordance with the severity of AEs. At the first occurrence of grade 3–4 symptoms, a dose reduction of 25% should be considered and maintained for the duration of treatment. At the second occurrence, a reduction to 50% is recommended, and a third occurrence of severe AE may warrant permanent treatment discontinuation. In a post hoc analysis of the ASCENT study, the efficacy outcomes for patients with dose reductions or interruptions in the SG arm were similar to those without dose reductions or interruptions [25].

## 4. Management of SG-Related Adverse Events

In the phase I/II IMMU-132-01 study, patients received optional premedications at the discretion of the treating physician. A total of 99% and 92% of patients in the 8 mg/kg and 10 mg/kg cohorts, respectively, experienced adverse events for all causalities, with a higher incidence of grade ≥3 events in the 10 mg/kg cohort [23]. To minimize adverse events and reduce discontinuation rates, pre-infusion strategies with concomitant medications and a standardized drug dose of 8 mg/kg were established in the subsequent ASCENT study. In the ASCENT study, a vast majority of participants, specifically 257 (99.6%) in the SG arm and 222 (99.1%) in the TPC arm, received pre-infusion and/or concomitant medications. The safety population included all participants who received a ≥1 dose of the study treatment in ASCENT. The pre-infusion and concomitant medication regimen primarily encompassed antiemetics, analgesics/antipyretics, antidiarrheals, and anti-inflammatory/antirheumatic agents. The percentage of patients in each arm who received these medications is detailed in Table 3 [20].

### 4.1. Neutropenia

Both neutropenia and diarrhea are toxicities associated with irinotecan, attributable to its active metabolite SN-38, which is the cytotoxic payload of SG [16]. In the ASCENT trial, the prevailing SG-related AE was neutropenia, documented in 63% of the SG arm and 43% of the TPC arm. The management for hematologic AEs included dose adjustments and the utilization of myeloid growth factors and blood transfusion in some patients [20]. Specifically, granulocyte colony-stimulating factor (G-CSF) was employed for 49% of those on the SG arm and 23% of those on the TPC arm. Notably, none of the participants in the SG arm had to discontinue treatment due to neutropenia-related concerns, while 1% in the TPC arm had to discontinue for this reason [20]. According to a post hoc safety analysis of the ASCENT study, the median time to onset for the first event of treatment-related grade ≥ 3 neutropenia was 21 days and 14 days for the SG and TPC arms, respectively. Moreover, the median duration for a single episode of grade ≥ 3 neutropenia was observed to be 6.0 days in the SG arm and 6.5 days in the TPC arm [25].

As per the trial protocol, G-CSF initiation was deemed appropriate based on clinical judgment and could be administered prophylactically, even as early as Cycle 1 [20]. G-CSF administration was as per the National Comprehensive Cancer Network^®^ (NCCN) guidelines. A prescribed daily dose of G-CSF at 5 μg/kg was recommended, continuing until a post-nadir absolute neutrophil count (ANC) recovery. In ASCENT, participants were required to have adequate cell counts without transfusion support, including an ANC > 1.5 × 10^9^/L [20]. The start of G-CSF was directed 24 h following the completion of chemotherapy, and its administration was to continue until the post-nadir recovery phase. To ensure appropriate management, the initiation of G-CSF therapy required an assessment of complete blood count (CBC) prior to the therapy’s commencement and through twice-weekly monitoring during the treatment course.

In Canada, the use of G-CSF in patients with metastatic disease is limited due to access and funding issues. To manage SG-related neutropenia in Canada, a combinational approach is required, with a mix of institutional/provincial guidelines and the ASCENT trial protocol recommended dose adjustments. Appendix A provides information regarding the provincial guidelines and manufacturers recommendations for SG-related AE in Canada. Treatment interruption or dose reduction is required for grade 3 or 4 events. If there are more than three prolonged grade 3 or 4 events, or if resolution takes more than 3 weeks, SG discontinuation is recommended. G-CSF support is suggested for secondary prophylaxis and can be used if available. For many cancer agencies in Canada, filgrastim is approved as primary prophylaxis, often in the curative setting for the prevention or mitigation of neutropenic complications in patients receiving an approved regimen, where the documented or expected incidence of febrile neutropenia has been identified as 20% or higher. Filgrastim is also approved at most agencies as secondary prophylaxis [34,35,36,37].

**Figure 1:**
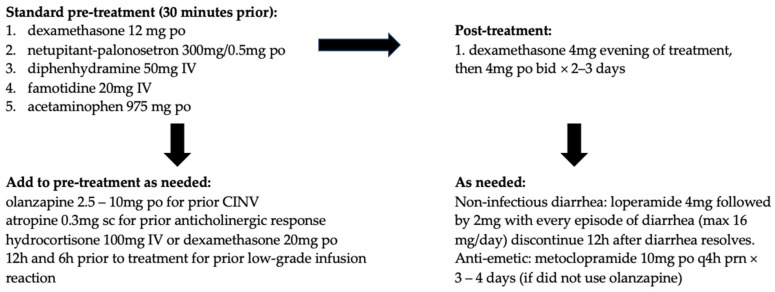
Authors’ recommended pre-treatment and concomitant medication regimen for sacituzumab govitecan (SG) based on information from the manufacturer, BC Cancer, Ontario Health/Cancer Care Ontario (OH/CCO), National Comprehensive Cancer Network^®^ (NCCN), and the Multinational Association of Supportive Care in Cancer (MASCC).

An ongoing multi-center phase II clinical trial (PRIMED) is evaluating the impact of primary prophylactic G-CSF for the management of neutropenia and primary prophylactic loperamide for the management of diarrhea. The co-primary endpoints of the study are the incidence of grade ≥ 3 neutropenia and grade ≥ 2 diarrhea after two treatment cycles of SG [38]. Preliminary data in 50 patients show a clinically relevant reduction in incidence as well as the severity of SG-related grade ≥ 3 neutropenia and grade ≥ 2 diarrhea with primary prophylactic administration of G-CSF at 0.5 MU/kg/day subcutaneously on cycle days 3, 4, 10, and 11, along with loperamide 2 mg twice a day or 4 mg once a day orally on cycle days 2, 3, 4, 9, 10, and 11 during at least the first two cycles [39]. Out of 50 participants in the PRIMED study, the incidence of any grade neutropenia (in the first two cycles) was 28%, compared to 63% and 70% in ASCENT and TROPiCS-02, respectively. The incidence of any grade diarrhea (in the first two cycles) was 34%, compared to 59% and 57% in ASCENT and TROPiCS-02, respectively. The smaller sample size in PRIMID may have contributed to the difference in reported neutropenia rates. Please see Figure 1 and Table 4 for author recommendations to manage SG-related AE based on guidelines from the manufacturer (Gilead), British Columbia Cancer Agency (BCCA), and Ontario Health/Cancer Care Ontario (OH/CCO); further information regarding these guidelines can be found in Appendix A.

### 4.2. Diarrhea

Diarrhea is likely attributable to the cytotoxic payload SN-38. Within the ASCENT safety population, 59% of those who received SG experienced any grade diarrhea, and 10% experienced grade 3. Comparatively, 12% had any grade diarrhea in the TPC arm and <1% experienced grade 3. Management includes dose interruption and potentially a dose reduction, as per the product monograph and provincial guidelines; further information is provided in Appendix A. The prompt initiation of loperamide is recommended if the etiology is not infectious. Fluid and electrolyte replacement may be given as supportive measures. SG discontinuation is recommended in those with three events of grade 3 or 4 diarrhea, or if resolution takes more than three weeks. For patients with excessive cholinergic symptoms (i.e., abdominal cramping, diarrhea, or salivation), treatment with atropine followed by prophylactic atropine use for subsequent SG infusions may be considered. As mentioned above, the PRIMED study showed clinical benefits with primary prophylactic loperamide in managing diarrhea with SG, and it can be considered an option at the discretion of the physician [39].

### 4.3. Nausea and Vomiting

As gastrointestinal toxicity may be limiting for SG, premedication for chemotherapy-induced nausea and vomiting (CINV) is highly recommended. Centers in Canada have varying premedication recommendations; for example, BCCA protocol labels SG as highly emetogenic, whereas OH/CCO labels it as moderate. SG is classified as highly emetogenic according to the NCCN, and therefore, a 2–3 drug antiemetic protocol is recommended, as demonstrated in Table 5 [40]. According to the Multinational Association of Supportive Care in Cancer (MASCC) guidelines, SG is labeled as moderately emetogenic with an asterisk, indicating it is high–moderate, resembling the carboplatin area under the curve (AUC) > 5 [41]; therefore, a three-drug regimen with dexamethasone, a 5-hydroxytryptamine (5HT3 or serotonin) receptor antagonist (RA), and a neurokinin 1 (NK1) RA is recommended [42]. In the ASCENT safety population, 57% of those who received SG experienced any grade nausea, as opposed to 26% in the TPC arm. Moreover, two percent of patients in the SG arm experienced grade 3 nausea and <1% were grade 4. In the TPC safety population, <1% experienced grade 3 nausea. Correspondingly, for vomiting, 29% of the SG safety population had any grade, with 1% experiencing grade 3 and <1% experiencing grade 4. In the TPC arm, 10% had any grade vomiting and <1% were grade 3. Overall, it is recommended to include a 2–3 drug CINV antiemetic protocol for patients receiving SG into the pre-treatment medication regimen and escalate as needed (the manufacturers’ recommendations, MASCC, and NCCN references are highlighted above). These protocols may include dexamethasone, a 5HT3 RA, and an NK-1 RA, and escalation may involve the addition of olanzapine. Refer to Figure 1 for the authors’ management recommendations [35,36,37,43,44].

### 4.4. Hypersensitivity and Skin Reactions

SG can cause life-threatening anaphylaxis and hypersensitivity reactions; therefore, it is recommended that patients receive antipyretics and antihistamines as part of the premedication regimen prior to all treatments [45]. A total of 34% of patients in the ASCENT study who received SG experienced hypersensitivity reaction of any grade within 24 h of SG dosing, and grade 3–4 hypersensitivity reactions occurred in 1.7% of patients treated with SG [46]. The incidence of a hypersensitivity reaction leading to the permanent discontinuation of SG was 0.1%. These rates highlight the need to incorporate additional mitigation measures into treatment protocols. As a further precaution, the first treatment can be delivered over an extended period of 3 h, with observation during and 30 min after infusion. If no hypersensitivity reactions are observed, further infusions can be administered over 1–2 h, with observation during and 30 min after. If a low-grade hypersensitivity reaction develops, recommendations are to slow or hold the infusion and add additional corticosteroids to future premedication regimens. If high-grade hypersensitivity reactions develop, it is recommended to stop the infusion, treat the reaction, and discontinue SG. Figure 1 depicts the authors’ recommendations for pre-treatment and concomitant medication regimen. Early signs of hypersensitivity could include skin reactions, with later and more severe signs presenting as angioedema, wheezing, pneumonitis, hypotension, other signs of anaphylaxis and ultimately cardiac arrest [45]. For these reasons, it is also recommended that emergency medications be available to treat severe hypersensitivity or anaphylaxis.

### 4.5. Alopecia

Alopecia of any grade was notably more frequent in the SG arm (46%) compared to TPC arm (16%) of the ASCENT study. Recently, scalp cooling with cold caps has been shown to reduce hair loss during chemotherapy administration in some centers [47,48]. However, the efficacy of cold capping in any ADC therapy specifically has not been reported.

### 4.6. UGT1A1 and Increased Risk of Adverse Events

Irinotecan metabolism occurs via UGT1A1-mediated glucuronidation. UGT1A1 is one of multiple splice variants derived from the UGT1A gene that is involved ubiquitously in the metabolic processing of a variety of lipophilic substrates [49]. Accordingly, the incidence of AEs, including neutropenia, anemia, and diarrhea, increases for patients who are homozygous for the UGT1A1*28 polymorphism [50]. The low-activity UGT1A1*28 allele is most frequent in Caucasian and African populations [50,51,52,53,54].

Of the combined 526 patients that were treated with SG between the ASCENT and TROPiCS-02 trials, 500 were screened for UGT1A1 genotypes. Within this cohort, 59 patients were homozygous for the UGT1A1*28 allele. Hematological AEs were notably increased in patients homozygous for the UGT1A1*28 allele [25,31]. As shown in Table 6, the incidence of grade 3–4 neutropenia varied among the three groups: 49.1% in wild-type homozygous, 52.6% in heterozygous, and 61% in homozygous mutation patients. Similarly, diarrhea was reported in 7.9% in wild-type homozygous, 11.2% in heterozygous, and 18.6% in homozygous patients. While these differences suggest a trend of increased adverse events in homozygous mutation patients, the variation in patient numbers between the groups limits the ability to draw definitive conclusions from these percentages. In Canada, screening for UGT1A1*28 allele is not standard procedure; however, genotyping may be considered in patients with severe AEs due to SG treatment.

### 4.7. Use in Special Populations

There are currently no clinical data available for SG treatment during pregnancy or for pediatric use in patients under 18 years of age. Of note, SG could cause embryo–fetal lethality when administered to a pregnant woman due to its mechanism of action and inherent toxicity to rapidly dividing cells [17,55]. Despite a lack of clinical data, manufacturers’ recommendations are to also advise against breastfeeding during and after SG treatment for a minimum of 1 month [17,55].

## 5. Discussion

The intended benefit of ADCs is tumor specificity, compared to standard chemotherapy regimens. Theoretically, this concentrated activity would be expected to expand the therapeutic index by both minimizing dosing requirements as well as off-target cytotoxic and adverse effects. However, many ADCs developed in recent years have demonstrated treatment-limiting toxicities [56]. The three ADCs approved for breast cancer indications discussed in this paper (T-DM1, T-DXd, and SG) are efficacious and have changed the breast cancer treatment landscape; however, they are not without significant toxicities. Given the poor outcomes for patients with aggressive diseases such as mTNBC, these AEs should be weighed along with the documented outcome benefits compared to the standard chemotherapy regimens currently in use. Recent findings have posited that the toxicities associated with ADCs are likely linked to the cytotoxic payload as opposed to their antigen targets [57]. Studies in which the ADC payload and linker remain consistent while the antibody of the ADC is varied demonstrate that AE incidence remains relatively consistent despite the potential for the distinct off-target binding effects of the antibody [56,58,59].

Many of the AEs reported with SG treatment can be managed with patient monitoring and appropriate interventions throughout the treatment and surveillance periods. Myelosuppression, diarrhea, nausea, fatigue, and alopecia should be highlighted for prospective patients as frequent but manageable toxicities. Within the ASCENT study, these events were most often recorded as lower grade (1 or 2); concurrent or pre-treatment with appropriate regimens can be considered at treatment onset to mitigate the impacts of these AEs. Neutropenia presents as one of the most significant toxicities associated with SG. Severe neutropenia may mandate delay, dose reductions, or the discontinuation of treatment. If access to G-CSF, a primary or secondary prophylaxis approach can be employed to manage SG-related neutropenia [34,35,36,37].

### Future Directions of SG

Given the improvements seen in metastatic breast cancer, SG is being studied in earlier stages of disease, including post-neoadjuvant HER2-negative breast cancer with residual invasive disease in the phase III SASCIA trial [60] and phase III ASCENT-05/OptimICE-RD trial [61], as well as neoadjuvant TNBC in the phase II NeoSTAR trial [62].

Finally, ADCs are being studied as combination regimens with traditional chemotherapy, endocrine therapy, targeted therapies, and immunotherapy. Theoretically, ADCs combined with immunotherapy may synergistically improve immune-mediated tumor destruction. In early-stage trials, the toxicity profiles are found to be additive and not synergistic. Regardless of additive toxicities, due to the compelling efficacy of enfortumab vedotin with pembrolizumab has been approved by the FDA for those with locally advanced or metastatic urothelial carcinoma who are ineligible for cisplatin-containing regimens [63]. SG is currently being studied in combination with pembrolizumab in the setting of untreated locally advanced inoperable or mTNBC with patients that are positive for PD-L1 in the phase III ASCENT-04 trial [64]. Additionally, SG is being studied as a monotherapy in the first-line metastatic setting for TNBC in the ASCENT-03 trial [32].

## 6. Conclusions

ADCs have revolutionized the treatment landscape for patients with mBC, early HER2-positive breast cancer, and many other solid tumors. SG is currently approved and funded in Canada for patients with mTNBC and for patients with HR-positive, HER2-negative unresectable locally advanced or mBC. Real-world experience with these novel agents and education regarding toxicity mitigation and management are crucial to ensuring patients derive maximum benefit from these therapies.

## Figures and Tables

**Table 1:** Comparison of efficacy data for patients with mTNBC treated with SG from the phase I/II basket study IMMU-132-01, phase III ASCENT, and phase III TROPiCS-02.

Efficacy Outcomes	IMMU-132-01 (n = 108) *	ASCENT (n = 235)	TROPiCS-02 (n = 272)
ORR	33%	35%	21%
Median PFS (95% CI), months	5.5 (4.1–6.3)	5.6 (4.3–6.3)	5.5 (4.2–7.0)
Median OS (95% CI), months	13.0 (11.2–13.7)	12.1 (10.7–14.0)	14.4 (13.0–15.7)
Median DOR (95% CI), months	9.1 (4.6–11.3)	6.3 (5.5–9)	8.1 (6.7–9.1)
CBR ^†^ (n) (95% CI)	45% (49) (35.8–55.2)	45% (105)	34% (92) (28.2–39.8)

* Only participants that received SG at 10 mg/kg were analyzed. ^†^ CBR was defined as participants who achieved CR + PR + SD for ≥6 months. BM, brain metastasis; CI, confidence interval; mTNBC, metastatic triple-negative breast cancer; ORR, objective response rate; OS, overall survival; PFS, progression-free survival; DOR, duration of response; CBR, clinical benefit rate; SG, sacituzumab govitecan.

**Table 2:** Treatment-related adverse events within the ASCENT study safety population.

Treatment-Related Adverse Event *	Sacituzumab Govitecan Percent of Total Treatment Population (n = 258)			Treatment of Physicians’ Choice Percent of Total Treatment Population (n = 224)		
	Any Grade	Grade 3	Grade 4	Any Grade	Grade 3	Grade 4
Any Adverse Event (%)	98	45	19	86	32	15
Hematologic						
Neutropenia	63	34	17	43	20	13
Anemia	34	8	0	24	5	0
Leukopenia	16	9	1	11	4	1
Thrombocytopenia	5	1	1	11	1	0
Febrile Neutropenia	6	5	1	2	2	<1
Gastrointestinal						
Diarrhea	59	10	0	12	<1	0
Nausea	57	2	<1	26	<1	0
Vomiting	29	1	<1	10	<1	0
Constipation	17	0	0	14	0	0
Abdominal Pain	11	1	0	4	<1	0
General						
Fatigue	45	3	0	30	5	0
Asthenia	12	1	0	10	1	0
Other						
Alopecia	46	0	0	16	0	0
Decreased appetite	20	2	0	14	<1	0
Nervous system disorders	25	<1	0	24	2	0
Respiratory, thoracic and mediastinal disorders	16	2	0	8	<1	0
Musculoskeletal and connective-tissue disorders	12	0	0	12	1	0
Infections and infestations	12	2	<1	10	2	1

* Shown are adverse events of any grade that occurred in at least 10% of the patients in either treatment group and adverse events of grade 3 or higher that occurred in at least 5% of the patients in either group.

**Table 3:** Percentage of patients in the ASCENT trial who received supportive medications by class.

Pre-Infusion or Concomitant Medications	Sacituzumab Govitecan	Treatment of Physicians’ Choice
Antiemetics/antinauseants	86%	63%
Analgesics/antipyretics	77%	64%
Antidiarrheals	55%	10%
Anti-inflammatory/anti-rheumatic agents	35%	33%
G-CSF	49%	23%

**Table 4:** Authors’ recommendations for the management of SG-related AEs. Absolute neutrophil count (ANC), serotonin receptor antagonist (5-HT3 RA), neurokinin-1 receptor antagonist (NK-1 RA), granulocyte colony-stimulating factor (G-CSF), chemotherapy-induced nausea vomiting (CINV), IRR, infusion-related reaction (IRR), intravenous (IV), administered orally (PO), subcutaneous (SC), hours (h), degree Celsius (°C).

Adverse Event	Authors’ Recommendations	
Severe Neutropenia	Occurrence	
Grade 4 neutropenia lasting ≥ 7 days OR Grade 3–4 febrile neutropenia OR At time of scheduled SG dose, grade 3–4 neutropenia that delays dosing by 2–3 weeks for recovery to grade ≤ 1	First	Reduce SG dose by 25% and administer G-CSF (consider filgrastim if available)
	Second	Reduce SG dose by 50% and administer G-CSF (consider filgrastim if available)
	Third	Discontinue SG and administer G-CSF (consider filgrastim if available)
At the time of scheduled SG dose, grade 3–4 neutropenia that delays SG dosing beyond 3 weeks for recovery to grade ≤ 1	First	Discontinue SG and administer G-CSF (consider filgrastim if available)
Non-Hematological Toxicities		
Diarrhea (grade 3 or 4) at the time of scheduled SG dose	Evaluate for infectious causes. If negative, initiate treatment. Treatment: At the onset of diarrhea, promptly initiate loperamide: ○ 4 mg initially, followed by 2 mg with every episode of diarrhea to a maximum of 16 mg daily; ○ Discontinue loperamide 12 h after diarrhea resolves. Supportive measures: Fluid and electrolyte replacement. Patient education: All patients should be given take-home medications with clear instructions for the treatment of diarrhea. Withhold SG until diarrhea is resolved to grade ≤ 1.	
Excessive cholinergic response (including abdominal cramping, diarrhea, rhinorrhea, increased salivation, lacrimation, diaphoresis, and flushing)	Provide atropine 0.3–0 6 mg IV or SC. Prophylactic atropine 0.3 mg SC prior to SG may be required for subsequent treatments.	
Nausea (grade 3) or vomiting (grade 3 or 4) at the time of scheduled SG dose	Premedication: A 2–3 drug combination regimen 30 to 60 min prior to SG infusion (e.g., dexamethasone with either a 5-HT3 RA or an NK-1 RA, as well as other drugs, as indicated) for the prevention of CINV. Consider dexamethasone 8 mg or 12 mg po, and the following: ○ Ondansetron 8 mg po 30 to 60 mg minutes prior to SG; ○ Aprepitant 125 mg po and ondansetron 8 mg IV or po; ○ Netupitant/palonosetron 300 mg–0.5 mg po. If additional antiemetics are required, consider olanzapine 2.5 mg or 5 mg po. Patient education: All patients should be given take-home medications with clear instructions for prevention and treatment of nausea/vomiting.	
Grade 4 diarrhea, nausea, or vomiting of any duration OR Grade 3 diarrhea, nausea, or vomiting not controlled by medication or persisting for >48 h despite optimal medical management OR At time of scheduled treatment, grade 3–4 non-neutropenic hematologic or non-hematologic toxicity which delays dose by 2–3 weeks for recovery to grade ≤ 1	First	Reduce SG dose by 25%
	Second	Reduce SG dose by 50%
	Third	Discontinue SG
Grade 3–4 non-neutropenic hematologic or non-hematologic toxicity which does not recover to grade ≤ 1 within 3 weeks	First	Discontinue SG
Hypersensitivity reaction	*Premedication:* Antipyretics and H1/H2 blockers prior to infusion. Corticosteroids may be used for patients who had prior infusion reactions prior to SG infusion. *Infusion:* The initial infusion should be slow (50 mg/h or less) and given over 3 h. If no IRR, subsequent infusions can be given over 1 h. *Monitoring:* monitor patients throughout and for ≥30 min post-infusion. Adjustment: ○ Grade 1: Slow the SG infusion. ○ Grade 2: Stop the SG infusion until symptoms resolve. Restart at a slower rate if the patient is stable. ○ Grade 3: Discontinue treatment.	
The dose of SG should not be re-escalated after a dose reduction for adverse reactions has been carried out		

**Table 5:** National Comprehensive Cancer Network^®^ (NCCN) recommended drug options based on category for highly emetogenic parenteral anticancer agents.

NCCN Preferred Options for Highly Emetogenic IV Agents		
Category	Day 1	Day 2, 3, 4
Corticosteroids	Dexamethasone 12 mg po/IV once	Dexamethasone 8 mg po/IV daily
5HT3 RA Choose 1	Granisetron 10 mg sc once, or 2 mg po once, or 0.01 mg/kg (max 1 mg) once, or 3.1 mg/24 h transdermal patch applied 24–48 h prior to first dose Ondansetron 16–24 mg po once or 8–16 mg IV once Palonosetron 0.24 mg IV once	
NK1 RA Choose 1	Aprepitant 125 mg po once Aprepitant injectable emulsion 130 mg IV once Fosaprepitant 150 mg IV once Netupitant 300 mg/palonosetron 0.5 mg po once	Aprepitant 80 mg po daily only if aprepitant po is used on day 1
Atypical Antipsychotic	Olanzapine 5–10 mg po once	Olanzapine 5–10 mg po daily

**Table 6:** (A) UGT1A1 allele status for patients in the SG arm of ASCENT, TROPiCS-02 and combined between these two studies. (B) Treatment-related adverse events and associated UGT1A1 allele status using combined data from patients in whom the UGT1A1 allele status is known in the SG arms of ASCENT and TROPiCS-02.

**(A)**			
**UGT1A1 Allele Status in SG Arm**	**ASCENT (%) (n = 258)**	**TROPiCS-02 (%) (n = 268)**	**Combined (%) (n = 526)**
UGT1A1 status known	250 (96.9)	250 (93.3)	500 (95.1)
Wildtype (*1/*1)	113 (43.8)	103 (38.4)	216 (41.1)
Heterozygous (*1/28)	96 (37.2)	119 (44.4)	215 (40.9)
Homozygous (*28/*28)	34 (13.1)	25 (9.3)	59 (11.2)
Other UGT1A1 genotype	7 (2.7)	3 (1.1)	10 (1.9)
**(B)**			
**Treatment-Related Adverse Event**	Homozygous Wild Type (*1/*1) (n = 216)	Heterozygous (*1/*28) (n = 215)	Homozygous (*28/*28) (n = 59)
All grade neutropenia	149 (69.0)	141 (65.6)	43 (72.9)
Grade 3 or 4 neutropenia	106 (49.1)	113 (52.6)	36 (61.0)
All grade anemia	71 (32.9)	72 (33.5)	28 (47.5)
Grade 3 or 4 anemia	11 (5.1)	16 (7.4)	7 (11.9)
All grade diarrhea	125 (57.9)	134 (62.3)	38 (64.4)
Grade 3 or 4 diarrhea	17 (7.9)	24 (11.2)	11 (18.6)

## Supplementary Materials

The following is available online at https://www.mdpi.com/article/10.3390/curroncol31090422/s1. File S1 Pharmacology Overview of SG.

## Appendix A

Links for Provincial and Manufacturers’ Guidelines:

1. British Columbia Cancer Agency Sacituzumab Govitecan protocol BRAVSG outlining SG for use as palliative therapy for metastatic triple negative breast cancer: http://www.bccancer.bc.ca/chemotherapy-protocols-site/Documents/Breast/BRAVSG_Protocol.pdf (accessed on 15 May 2024).
2. Cancer Care Ontario Drug Formulary link for Sacituzumab Govitecan: https://www.cancercareontario.ca/en/drugformulary/drugs/monograph/74306 (accessed on 20 February 2024).
3. Gilead product monograph for Trodelvy (Sacituzumab Govitecan): https://pdf.hres.ca/dpd_pm/00071772.PDF (accessed on 16 May 2024).
