# Supplementary material for: Canadian Expert Recommendations on Safety Overview and Toxicity Management Strategies for Sacituzumab Govitecan Based on Use in Metastatic Triple-Negative Breast Cancer
Source: Curr Oncol. 2024 Sep 21;31(9):5694–708. doi: 10.3390/curroncol31090422 (PMC11431578; doi:10.3390/curroncol31090422)
Supplement: Supplementary file 1 [file curroncol-31-00422-s001.zip › curroncol-3176430-supplementary.pdf]

SUPPLEMENTARY MATERIAL

1. Pharmacology Overview of SG

The PK profile of SG in participants with advanced solid tumors was characterized using concentration-time data from the IMMU-132-01 [23], TROPHY-U-01 [24], and ASCENT [25,26] studies. The PK of SG, free SN-38, total SN-38, total antibody, and the glucuronide metabolite of free SN-38 (SN-38G) were assessed following administration of SG 10 mg/kg on days 1 and 8 of a 21-day treatment cycle. No accumulation of SG or free SN-38 was observed after multiple cycles. Across the studies, SG and free SN-38 exposures were comparable (Table 2). The mean clearance and steady-state volume of distribution for SG ranged from 0.14 to 0.16 L/h and from 2.45 to 2.82 L, respectively [27].

PK Parameter	IMMU-132-01 (275)			TROPHY-U-01 (131)			ASCENT (253)		
	SG	Free SN-38	Total Antibody	SG	Free SN-38	Total Antibody	SG	Free SN-38	Total Antibody
C_{max}, mean (%CV), ng/mL	227,000 (24)	120 (82)	252,000 (27)	224,000 (23)	67.3 (44)	228,000 (23.8)	240,000 (22)	90.6 (65)	281,000 (39.1)
T_{max}, median (range), h	3.03 (1.1-23.3)	3.58 (1.5-6.7)	3.42 (2.5-24.9)	3.52 (1.6-4.6)	4.6 (3-7.2)	4.67 (3-21.3)	3.09 (1.2-5.4)	3.25 (1.2-6.3)	3.07 (2.6-6.1)
$AUC_{0–168}$ h, mean (%CV), ng·h/mL	5,190,000 (24)	3620 (72)	21,200,000 (21.4)	5,270,000 (31)	1970 (37)	21,000,000 (32.2)	5,340,000 (24)	2730 (41)	18,100,000 (20.5)
$t_{½}$, median (range), h	14.7 (12.1-27.5)	16.9 (11.5-30.3)	63.1 (57.4-83.4)	14 (12–15.4)	16.2 (13.6-23.4)	122 (56.1-279)	14.7 (8.83-24.7)	17.6 (11.1-44)	60.1 (9.05-97.6)
V_{ss},[a] L	2820	6900	-	-	-	-	-	-	-
C_L,[a] L/h	149	270,000	-	-	-	-	-	-	-

C_L=total body clearance; T_{max}=time to maximum concentration; Vss=apparent volume of distribution at steady state following IV administration. [a]Measure (eg, mean, median) was not specified in the source.

Table 1. Pharmacokinetic parameters for sacituzumab govitecan (SG), free SN-38, and total antibody after the first dose of SG 10 mg/kg in the IMMU-132-01, TROPHY-U-01, and ASCENT Studies [5].

An analysis of the uridine diphosphate glucuronosyltransferase family 1 member A1 (UGT1A1) polymorphisms in the IMMU-132-01, TROPHY-U-01, and ASCENT studies showed no impact on the PK of SG or free SN-38, even with concomitant UGT1A1 inhibitors or inducers. The population PK model indicated that UGT1A1 genotype was not a significant factor for AUG or Cmax of SG or free SN-38. Additionally, these studies found no clinically relevant changes in SG exposure related to renal or hepatic impairment, age, sex, albumin level, race, Eastern Cooperative Oncology Group (ECOG) performance status, tumor type, or Trop-2 expression [27].

References

23. Bardia, A.; Messersmith, W. A.; Kio, E. A.; Berlin, J. D.; Vahdat, L.; Masters, G. A.; Moroose, R.; Santin, A. D.; Kalinsky, K.; Picozzi, V.; et al. Sacituzumab Govitecan, a Trop-2-Directed Antibody-Drug Conjugate, for Patients with Epithelial Cancer: Final Safety and Efficacy Results from the Phase I/II IMMU-132-01 Basket Trial. *Annals of Oncology*, **2021**, *32* (6), 746–756. https://doi.org/10.1016/j.annonc.2021.03.005.
24. Tagawa, S. T.; Balar, A. V; Petrylak, D. P.; Rezazadeh Kalebasty, A.; Loriot, Y.; Fí echon, A.; Jain, R. K.; Agarwal, N.; Bupathi, M.; Barthelemy, P.; et al. *A Phase II Open-Label Study of Sacituzumab Govitecan in Patients With*

Metastatic Urothelial Carcinoma Progressing After Platinum-Based Chemotherapy and Checkpoint Inhibitors; 2021; Vol. 39.
25. Rugo, H. S.; Tolaney, S. M.; Loirat, D.; Punie, K.; Bardia, A.; Hurvitz, S. A.; O'Shaughnessy, J.; Cortés, J.; Diéras, V.; Carey, L. A.; et al. Safety Analyses from the Phase 3 ASCENT Trial of Sacituzumab Govitecan in Metastatic Triple-Negative Breast Cancer. *NPJ Breast Cancer*, **2022**, *8* (1), 98. https://doi.org/10.1038/s41523-022-00467-1.
26. Hurvitz, S. A.; Bardia, A.; Punie, K.; Kalinsky, K.; Cortés, J.; O'Shaughnessy, J.; Carey, L. A.; Rugo, H. S.; Yoon, O. K.; Pan, Y.; et al. 168P Sacituzumab Govitecan (SG) Efficacy in Patients with Metastatic Triple-Negative Breast Cancer (MTNBC) by HER2 Immunohistochemistry (IHC) Status: Findings from the Phase III ASCENT Study. *Annals of Oncology*, **2022**, *33*, S200–S201. https://doi.org/10.1016/j.annonc.2022.03.187.
27. Sathe, A. G.; Singh, I.; Singh, P.; Diderichsen, P. M.; Wang, X.; Chang, P.; Taqui, A.; Phan, S.; Girish, S.; Othman, A. A. Population Pharmacokinetics of Sacituzumab Govitecan in Patients with Metastatic Triple-Negative Breast Cancer and Other Solid Tumors. *Clin Pharmacokinet*, **2024**, *63* (5), 669–681. https://doi.org/10.1007/s40262-024-01366-3.
